# Intestinal Microbiome‐Macrophage Crosstalk Contributes to Cholestatic Liver Disease by Promoting Intestinal Permeability in Mice

**DOI:** 10.1002/hep.31228

**Published:** 2020-12-23

**Authors:** Anna Isaacs‐Ten, Marta Echeandia, Mar Moreno‐Gonzalez, Arlaine Brion, Andrew Goldson, Mark Philo, Angela M. Patterson, Aimee Parker, Mikel Galduroz, David Baker, Simon M. Rushbrook, Falk Hildebrand, Naiara Beraza

**Affiliations:** ^1^ Gut Microbes and Health Institute Strategic Programme Quadram Institute Bioscience Norwich Research Park Norwich United Kingdom; ^2^ Analytical Science Unit Quadram Institute Bioscience Norwich Research Park Norwich United Kingdom; ^3^ Science Operations Quadram Institute Bioscience, Norwich Research Park Norwich United Kingdom; ^4^ Department of Gastroenterology Norfolk and Norwich University Hospital Norwich United Kingdom; ^5^ Digital Biology Earlham Institute Norwich United Kingdom; ^6^ Food Innovation and Health Institute Strategic Programme Quadram Institute Bioscience Norwich Research Park Norwich United Kingdom

## Abstract

**Background and Aims:**

Mounting evidence supports an association between cholestatic liver disease and changes in the composition of the microbiome. Still, the role of the microbiome in the pathogenesis of this condition remains largely undefined.

**Approach and Results:**

To address this, we have used two experimental models, administering alpha‐naphtylisocyanate or feeding a 0.1% 3,5‐diethoxycarbonyl‐1,4‐dihydrocollidine diet, to induce cholestatic liver disease in germ‐free mice and germ‐free mice conventionalized with the microbiome from wild‐type, specific pathogen‐free animals. Next, we have inhibited macrophage activation by depleting these cells using clodronate liposomes and inhibiting the inflammasome with a specific inhibitor of NOD‐, LRR‐, and pyrin domain‐containing protein 3. Our results demonstrate that cholestasis, the accumulation of bile acids in the liver, fails to promote liver injury in the absence of the microbiome *in vivo*. Additional *in vitro* studies supported that endotoxin sensitizes hepatocytes to bile‐acid–induced cell death. We also demonstrate that during cholestasis, macrophages contribute to promoting intestinal permeability and to altered microbiome composition through activation of the inflammasome, overall leading to increased endotoxin flux into the cholestatic liver.

**Conclusions:**

We demonstrate that the intestinal microbiome contributes to cholestasis‐mediated cell death and inflammation through mechanisms involving activation of the inflammasome in macrophages.

**Abbreviations**
AKT: protein kinase B
ANIT: alpha‐naphtylisocyanate
AP: alkaline phosphatase
Bsep: bile salt export pump
CA: cholic acid
CDCA: chenodeoxycholic acid
Cyp2b10: cytochrome P450, family 2, subfamily b, polypeptide 10
Cyp2c70: cytochrome P450, family 2, subfamily c, polypeptide 70
Cyp3a11: cytochrome P450, family 3, subfamily a, polypeptide 11
DCA: deoxycholic acid
DDC: 3,5‐diethoxycarbonyl‐1,4‐dihydrocollidine
FC: Flow cytometry
FITC: fluorescein isothiocyanate
GCA: glycocholic acid
GF: germ‐free
Gst3: glutathione S‐transferase 3
Gxp1: glutathione peroxidase 1
HPLC: high‐performance liquid chromatography
IBD: inflammatory bowel disease
IL1β: interleukin‐1 beta
LPS: lipopolysaccharide
MCA: murocholic acid
Mdr2: multidrug resistance protein 2
Mrp2: multidrug resistance‐associated protein 2
Mrp4: multidrug resistance‐associated protein 4
MS: mass spectrometry
Nlrp3: NOD‐, LRR‐, and pyrin domain‐containing protein 3
Ntcp: Na+‐taurocholate cotransporting polypeptide
Oatp: organic anionic transporting polypeptide
PBC: primary biliary cholangitis
PSC: primary sclerosing cholangitis
rRNA: ribosomal RNA
T‐β‐MCA: tauro beta murocholic acid
TCA: taurocholic acid
TJ: tight junction
TNF: tumor necrosis factor
Ugt1a1: UDP glucuronosyltransferase family 1 member A1
Ugt1a2: UDP glucuronosyltransferase family 1 member A2
WT: wild type

The intestine is a selective barrier that prevents pathogenic bacteria translocating to the systemic circulation, while simultaneously allowing nutrient absorption. In the intestine, crosstalk regulation among the microbiome, the immune system, and epithelial cells is essential to preserve barrier function.^(^ ^1^, ^2^ ^)^ Intestinal permeability is tightly regulated by the immune system as inflammatory cytokines (e.g., tumor necrosis factor [TNF] and interferon gamma) modulate the expression of tight junction (TJ) proteins.^(^ ^3^ ^)^ In the intestine, the microbiome shapes the immune system, rendering a tolerant environment where microbes and host cells can coexist.^(^ ^1^, ^2^ ^)^ Reciprocally, inflammation can determine the composition of the intestinal microbiome,^(^ ^4^, ^5^ ^)^ adding another layer of complexity to the regulation of intestinal permeability and barrier function.

The “leaky gut” hypothesis proposes that chronic liver disease is associated with breaching of the intestinal barrier attributed to increased permeability allowing bacterial translocation into the liver. There, microbe‐derived products are recognized by the innate immune system through pathogen recognition receptors (Toll‐like receptors and Nod‐like receptors), leading to activation of the inflammasome and proinflammatory cytokine production.^(^ ^6^ ^)^ When liver inflammation persists unresolved, the proinflammatory milieu has detrimental effects on parenchymal and nonparenchymal liver cells, leading to fibrosis and, ultimately, loss of function.^(^ ^7^, ^8^ ^)^

Cholestasis occurs when the flow of bile from the liver to the small intestine is impaired, which is a common secondary pathological feature of chronic liver diseases.^(^ ^9^ ^)^ In adults, the main etiologies of cholestasis are primary biliary cholangitis (PBC) and primary sclerosing cholangitis (PSC), where tissue damage is typically linked to inflammation that triggers bile duct destruction and fibrosis.^(^ ^10^, ^11^ ^)^

Recent studies have demonstrated that human cholestasis is associated with changes in microbiome composition. The microbiome in PSC patients (with or without inflammatory bowel disease [IBD]) is distinct from the microbiome of patients with IBD only; less diverse and enriched with *Enterococcus* and *Lactobacillus* in fecal samples^(^ ^12^, ^13^, ^14^ ^)^ and enriched with *Escherichia* and *Clostridiales* species in mucosal samples.^(^ ^15^ ^)^ Some of these changes observed in the microbiome of PSC patients also occur in PBC, including reduced diversity and increased abundance of *Streptococcus* and *Lactobacillus* species.^(^ ^16^ ^)^

In addition to changes in microbiome composition, increased permeability has been described in PBC patients,^(^ ^17^, ^18^ ^)^ and the close association of PSC with IBD^(^ ^19^ ^)^ overall supports the hypothesis that translocation of bacterial products from the leaky gut may relate to the pathogenesis of cholestatic disease.

Our work provides fundamental knowledge on the mechanisms mediating the pathogenesis of cholestatic liver disease. Our results demonstrate that the microbiome actively contributes to liver injury during cholestasis and provide mechanistic evidence on the detrimental role of macrophages in contributing to cholestasis‐mediated liver injury by promoting intestinal permeability and altering the intestinal microbiome composition.

## Materials and Methods

### Experimental Procedures in Animals

Germ‐free (GF) mice were generated on a *C57/B6J* background in the Disease Modelling Unit (University of East Anglia, UK). A subset of GF mice was conventionalized with the microbiome obtained from specific pathogen‐free (SPF) wild‐type (WT) mice by administration of fecal matter by gavage 3 weeks before initiation of the experiments (GF + WT).

Cholestasis was induced by administration of alpha‐naphtylisocyanate (ANIT) for 48 hours and by feeding a subset of mice with a diet containing 3,5‐diethoxycarbonyl‐1,4‐dihydrocollidine (DDC) for 1 week as we described.^(^ ^20^ ^)^ GF + WT mice were treated either with vehicle (corn oil) or ANIT (100 mg/kg) by oral gavage, whereas a subset of GF + WT mice were pretreated with clodronate (10 mL/kg) by intraperitoneal injection 24 hours before, at the time of the administration of ANIT, and 24 hours after. The NOD‐, LRR‐, and pyrin domain‐containing protein 3 (Nrlp3)‐specific inhibitor, MCC950 (20 mg/kg), was administered to GF + WT mice in parallel to the administration of ANIT and 24 hours after. All ANIT‐treated animals were euthanized 48 hours after gavage.

All experimental procedures were conducted in male mice from 8 to 12 weeks of age that were held in isolators in the germ‐free facility at the Disease Modelling Unit (University of East Anglia, UK). All experiments were previously approved by the Animal Welfare and Ethical Review Body (University of East Anglia, Norwich, UK) and were performed following the guidelines of the National Academy of Sciences (National Institutes of Health publication 86‐23, revised 1985) and were conducted within the provisions of the Animals (Scientific Procedures) Act (1986) and the LASA Guiding Principles for Preparing for and Undertaking Aseptic Surgery (2010) under UK Home Office approval (70/8929).

### Statistical Analysis

Data are expressed as mean ± standard error of the mean. Statistical significance was determined by two‐way analysis of variance and by a Student’s t test when appropriate. 16s rRNA sequencing data analysis was conducted with R statistical language Version 3.00 (The R Foundation, https://www.r-project.org/) as described in Hildebrand et al.,^(^ ^21^ ^)^ employing the rtk software^(^ ^22^ ^)^ or all data normalizations.

More information can be found in the Supplemental Material and Methods.

## Results

### The Absence of the Intestinal Microbiome Protects the Liver From Injury and Inflammation During Cholestasis

We induced cholestasis in GF mice, lacking a microbiome, and in GF mice conventionalized with the microbiota obtained from SPF WT mice (GF + WT) using two experimental models; the administration of ANIT and feeding with a diet containing 0.1% DDC.

**Fig. 1:**
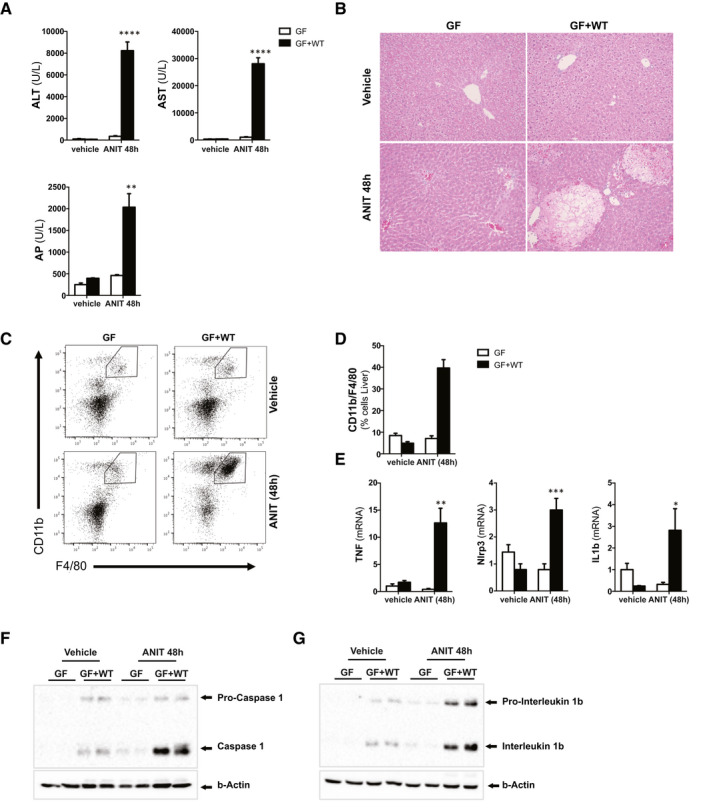
Absence of a microbiome protects mice from cholestatic‐induced liver injury and inflammation. (A) Levels of blood liver injury and cholestasis markers and (B) H&E staining of liver sections from GF and GF mice conventionalized with WT microbiome (GF + WT) treated with vehicle or ANIT (100 mg/kg) for 48 hours. (C) FC analysis on liver‐isolated immune cells, (D) further quantification, and (E) qPCR analysis on liver extracts showing increased presence of macrophages and inflammation in ANIT/GF + WT mice. (F, G) Western blotting analysis of whole‐liver lysates showing activation of the inflammasome. Images are representative of n ≥ 5 animals per treatment group. Values are mean ± SEM. *P < 0.05; **P < 0.01; ***P < 0.001 (GF vs. GF+WT). Abbreviations: ALT, alanine aminotransferase; AST, aspartate aminotransferase; FC, flow cytometry; H&E, hematoxylin and eosin.

Sterile GF mice treated with ANIT for 48 hours showed low levels of liver‐damage and cholestasis‐serum markers and the virtual absence of necrotic areas in the liver (Fig. 1A,B). In contrast, conventionalized GF + WT showed severe liver injury after ANIT treatment as evidenced by high levels of serum transaminases and alkaline phosphatase (AP) and wide areas of necrosis throughout the liver parenchyma (Fig. 1A,B)

The dramatic worsening of the liver phenotype in conventionalized GF + WT mice after ANIT treatment was associated with increased inflammation, as shown by the significant increase of infiltrating macrophages in the liver (Fig. 1C, D) and expression of proinflammatory cytokines (Fig. 1E) in conventionalized GF + WT mice compared to GF animals.

Macrophages represent the first line of defense against invading bacteria, promoting activation of the inflammasome, triggering the interleukin‐1 beta (IL1β)‐dependent proinflammatory response by cleavage of pro‐IL1β by caspase 1.^(^ ^23^, ^24^ ^)^ Our results demonstrate that conventionalization with a WT microbiome promoted a mild activation of the inflammasome in vehicle/GF + WT mice, which was further exacerbated after ANIT treatment given that we found high levels of cleaved caspase 1 and cleaved IL1β in comparison to GF mice (Fig. 1F,G).

**Fig. 2:**
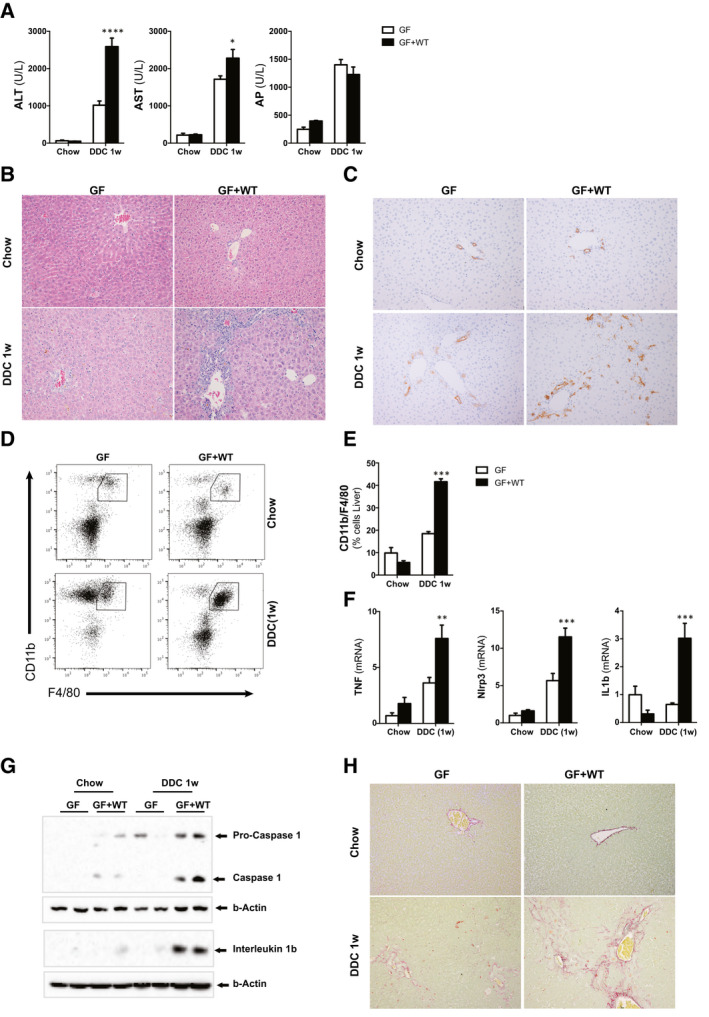
Absence of a microbiome protects mice from 0.1% DDC diet–induced liver injury and inflammation. (A) Levels of blood liver injury and cholestasis markers. (B) H&E staining of liver sections from GF and GF mice conventionalized with WT microbiome (GF + WT) fed with chow or 0.1% DDC diet for 1 week. (C) IHC using an anti‐CK19 Ab in paraffin‐embedded liver sections showing milder ductular reaction in GF mice compared to GF + WTs. (D) FC analysis on liver‐isolated immune cells, (E) further quantification, and (F) qPCR analysis on liver extracts showing increased presence of macrophages and inflammation in 0.1% DDC/GF + WT mice. (G) Western blotting analysis of whole‐liver lysates showing activation of the inflammasome. (H) Liver fibrosis was assessed by Sirius Red staining on liver sections. Images are representative of n ≥ 5 animals per treatment group. Values are mean ± SEM. *P < 0.05; **P < 0.01; ***P < 0.001 (GF vs. GF + WT). Abbreviations: Ab, antibody; ALT, alanine aminotransferase; AST, aspartate aminotransferase; FC, flow cytometry; H&E, hematoxylin and eosin; IHC, immunohistochemistry.

Further experiments in mice fed with a 0.1% DDC diet for 1 week^(^ ^20^ ^)^ confirmed the detrimental impact of the intestinal microbiome in the cholestatic liver. Sterile 0.1% DDC/GF mice showed significantly lower serum transaminases, but similar AP levels, compared to 0.1% DDC/GF + WT mice, which exhibited histological features characteristic of cholestatic disease including ductular reaction and areas of tissue damage (Fig. 2A,B). Immunohistochemical analysis confirmed significantly fewer cytokeratin 19 (CK19)‐positive cells in GF mice when compared to GF + WT mice after 0.1% DDC treatment (Fig. 2C and Supporting Fig. S1A).

The worsening on the liver phenotype in conventionalized GF + WT mice after the 0.1% DDC diet was associated with increased inflammation, as evidenced by higher infiltration of macrophages (Fig. 2D,E), expression of proinflammatory markers (Fig 2F), and activation of the inflammasome (Fig. 2G) when compared to GF mice.

GF mice had significantly reduced fibrosis when compared to conventionalized GF + WT mice, which showed higher collagen deposition in the liver after the 0.1% DDC diet as evidenced by Sirius Red staining (Fig. 2H), and increased collagen and alpha‐smooth muscle actin gene expression (Supporting Fig. S1B).

Overall, our results show that the microbiome exacerbates the progression of cholestatic liver disease.

### Cholestasis Occurs Independently of the Presence of the Microbiome That Influences the Bile Acid Pool Composition Before and After ANIT and 0.1% DDC Treatment

The significant protection from cholestatic liver injury shown by mice lacking a microbiome could arguably reflect a lower degree of cholestasis, or result from a different bile acid metabolism and pool composition compared to GF + WT mice after ANIT and 0.1% DDC treatments.

**Fig. 3:**
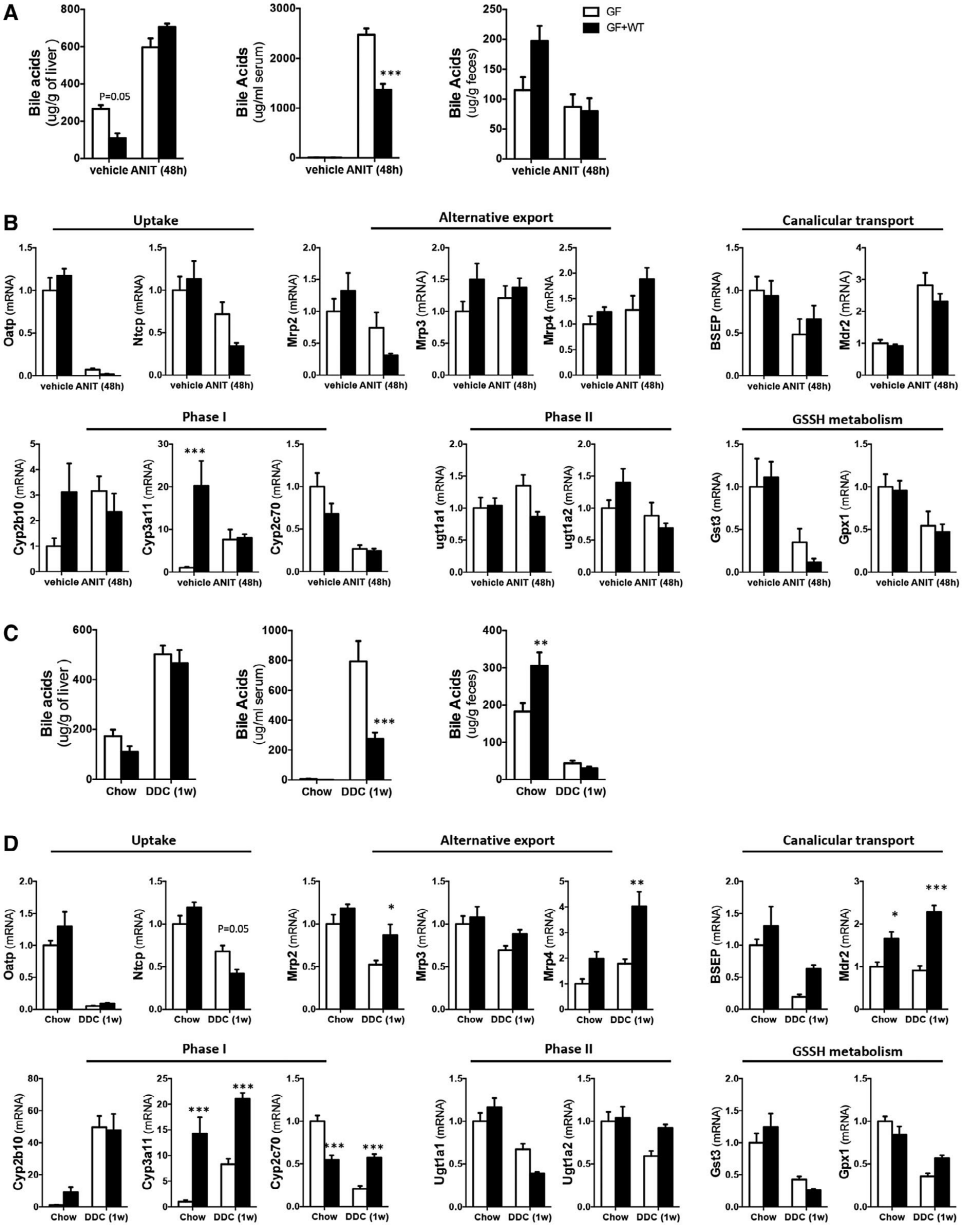
ANIT and 0.1% DDC promote similar cholestasis in GF and GF + WT mice while showing differences in bile acid metabolism. (A) Quantification of bile acid pool size in livers, serum, and fecal samples from WT and GF + WT mice by MS‐HPLC at 48 hours after ANIT. (B) Gene expression determined by qPCR of xenobiotic and bile acids transporters, phase I and II detoxification, as well as glutathione metabolism in liver samples from GF and GF + WT mice after ANIT. (C) Quantification of bile acid pool size in livers, serum, and fecal samples from WT and GF + WT mice by MS‐HPLC after feeding with 0.1% DDC for 1 week. (D) qPCR of xenobiotic and bile acids metabolism in liver samples from GF and GF + WT mice after 0.1% DDC. Graphs show results from n ≥ 5 animals per treatment group. Values are mean ± SEM. *P < 0.05; **P < 0.01; ***P < 0.001 (GF vs. GF + WT). Abbreviations: GSSH, oxidized glutathione; Mrp3, multidrug resistance‐associated protein 3.

Bile acid pool size was determined in liver, serum, and fecal samples using high‐performance liquid chromatography (HPLC)/mass spectrometry (MS). In agreement with previous studies,^(^ ^25^ ^)^ GF mice accumulated more bile acids in the liver while having secreted less in serum and feces at basal conditions (Fig. 3A; Supporting Tables S1‐S3). After ANIT, GF and GF + WT mice showed elevated and comparable accumulation of bile acids in livers (Fig. 3A). Consistently with the induction of cholestasis, we found elevated presence of bile acids in serum in both groups that was significantly higher in ANIT/GF mice compared to ANIT/GF + WT animals, whereas fecal levels of bile acids were reduced and reached similar levels in both ANIT‐treated groups (Fig. 3A).

Bile acid pool composition in livers after ANIT treatment was dominated by tauro beta (T‐β‐MCA) murocholic acid (MCA) similarly in both groups, with tauro alpha MCA and taurocholic acid (TCA) being predominant in GF + WT while β‐MCA and tauroursodeoxycholic acid were more abundant in GF mice (Supporting Table S1). In serum samples, secretion of TCA was comparable whereas ANIT/GF mice showed significantly more T‐β‐MCA and β‐MCA than ANIT/GF + WT animals (Supporting Table S2). Fecal bile acids were predominantly T‐β‐MCA and TCA in ANIT/GF whereas ANIT/GF + WT mainly secreted cholic acid (CA) and deoxycholic acid (DCA) in agreement with the presence of a microbiome (Supporting Table S3).

A comparable trend was found in livers, serum, and fecal samples after 0.1% DDC treatment (Fig. 3C), although the differences in bile acid composition were less pronounced between GF and GF + WT mice, with the exception of T‐β‐MCA, which was predominantly found in serum and feces from 0.1% DDC/GF mice (Supporting Tables S4‐S6).

Analysis of the hepatic metabolism of xenobiotics and bile acids confirmed the expected down‐regulation of the uptake transporter, organic anionic transporting polypeptide (Oatp), after ANIT^(^ ^26^ ^)^ that was comparable in GF and GF + WT mice, whereas the reduction in Na^+^‐taurocholate cotransporting polypeptide (Ntcp) expression was more pronounced in GF + WT animals (Fig. 3B). Canalicular export genes were similarly regulated in GF and GF + WT mice, and the mild differences in alternative transporter expression did not reach statistical significance (Fig. 3B). Phase I enzymes cytochrome P450, family 2, subfamily b, polypeptide 10 (Cyp2b10) and cytochrome P450, family 3, subfamily a, polypeptide 11 (Cyp3a11) were reduced whereas cytochrome P450, family 2, subfamily c, polypeptide 70 (Cyp2c70) was elevated in GF versus GF + WT mice at basal conditions. After ANIT treatment, all were significantly decreased and reached comparable levels in ANIT/GF versus ANIT/GF + WT mice (Fig. 3B). Expression of glucuronidation‐related enzyme UDP glucuronosyltransferase family 1 member A1 (Ugt1a1) remained elevated in GF mice whereas it was mildly reduced in GF + WTs (Fig. 3B). UDP glucuronosyltransferase family 1 member A2 (Ugt1a2) was increased basally in GF + WT mice and was similarly reduced in both groups after ANIT (Fig. 3B). Last, glutathione *S*‐transferase 3 (Gst3) expression was mildly higher in GF mice compared to GF + WT whereas no differences were found in glutathione *S*‐transferase 4 (Gst4; data not shown) and glutathione peroxidase 1 (Gxp1) after ANIT (Fig. 3B).

In 0.1% DDC–treated mice, we found decreased uptake (Oatp, Ntcp), as expected,^(^ ^27^ ^)^ while Ntcp remained higher in GF compared to GF + WT mice (Fig. 3D). Increased basolateral (multidrug resistance‐associated protein 2 [Mrp2], multidrug resistance‐associated protein 4 [Mrp4]) and canalicular transport (bile salt export pump [Bsep] and multidrug resistance protein 2 [Mdr2]) were found in GF + WT mice compared to GF (Fig. 3D), though these changes did not prevent the increase in circulating bile acids in 0.1% DDC/GF mice (Supporting Table S5). Phase I detoxification enzymes Cyp3a11 and Cyp2c70 were elevated in GF + WT animals, and phase II and glutathione‐metabolism–related genes were differentially regulated in both groups (Fig. 3D).

Although the microbiome influences bile acid hydroxylation and moderately impacts on bile acid transporter expression, overall, our results show that cholestasis, the accumulation of bile acids in the liver and serum, occurs independently of the presence of the intestinal microbiome.

### Bile‐Acid–Induced Cell Death Is Reduced in Hepatocytes From Germ Free Mice

To determine whether the liver protection observed in GF mice could result from the different bile acid pool composition (Supporting Tables S1‐S6), we performed *in vitro* studies where we exposed hepatocytes isolated from GF and conventionalized GF + WT mice to bile acids.

**Fig. 4:**
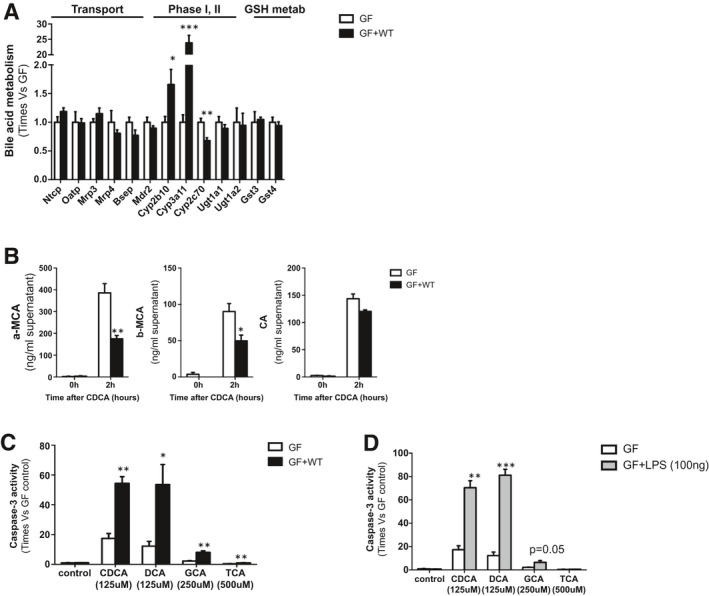
Endotoxin sensitizes GF hepatocytes to bile‐acid–induced cell death. (A) qPCR to determine the expression of xenobiotic and bile acid transporters, phase I and phase II detoxification, as well as glutathione metabolism at basal conditions in cultured hepatocytes isolated from GF and GF + WT mice. (B) Quantification of α‐MCA, β‐MCA, and CA in the supernatants of cultured hepatocytes 2 hours after CDCA (125 μM) and DCA (125 μM) stimulation. (C) Caspase 3 activity was determined in isolated hepatocytes from GF and GF + WT mice 2 hours after CDCA (125 μM), DCA (125 μM), GCA (250 μM), and TCA (500 μM) and in hepatocytes (D) pretreated for 4 hours with LPS (100 ng/mL). Values are mean ± SEM. *In vitro* experiments were done twice in triplicate. *P < 0.05; **P < 0.01; ***P < 0.001 (GF vs. GF + WT). Abbreviations: GSH, glutathione; Mrp3, multidrug resistance‐associated protein 3.

We confirmed that cultured GF and GF + WT hepatocytes retained a similar expression pattern of uptake, alternative, and canalicular transporters as well as a lower Cyp2b10 and Cyp3a11, with higher Cyp2c70 expression in GF hepatocytes (Fig. 4A). MS analysis showed that the conversion rate of chenodeoxycholic acid (CDCA) into MCA species was increased in GF hepatocytes whereas the conversion of DCA into CA was comparable in GF and GF + WT hepatocytes (Fig 4B). These results suggest that the hydroxylating capacity of GF hepatocytes could be mediated by Cyp2c70 and/or (the residual) Cyp2b10 independently of Cyp3a11. This is in line with previous work showing that Cyp3a11 is dispensable for bile acid detoxification.^(^ ^28^ ^)^

We then determined the impact of different bile acids in hepatocyte viability and found lower caspase 3 activity, indicative of cell death by apoptosis, in GF hepatocytes, as opposed to GF + WT hepatocytes, that showed profuse apoptotic cell death after CDCA, DCA, and glycocholic acid (GCA) and, to a lower degree, after TCA (Fig. 4C). Microscopic observation of cell cultures confirmed increased cell death in GF + WT mice compared to GF cells after bile acids (Supporting Fig. S2A).

Ultimately, to confirm our hypothesis that bacterial products contribute to bile‐acid–mediated cell death, we pretreated GF hepatocytes with lipopolysaccharide (LPS). This treatment significantly sensitized GF hepatocytes to bile‐acid–induced cell death that evidenced a dramatic increase in caspase 3 activity, particularly in LPS/CDCA‐ and LPS/DCA‐treated hepatocytes (Fig. 4D). The significant impact of bile acids in promoting cell death in LPS/GF hepatocytes occurred independently of the detoxification capacity of these cells. Thus, whereas LPS reduced the intracellular presence of α‐MCA (though not significantly), β‐MCA and CA levels remained comparable to those found in GF mice (Supporting Fig. S2B).

Overall, our *in vitro* results confirmed that exposure to bacterial endotoxin sensitizes hepatocytes to bile‐acid–induced cell death.

### The Intestinal Microbiome Contributes to Increase Intestinal Permeability and Inflammation During Cholestasis

Based on the leaky gut hypothesis, cholestasis is associated with increased intestinal permeability, allowing the translocation of bacterial products into the liver, where they contribute to injury and disease progression.

**Fig. 5:**
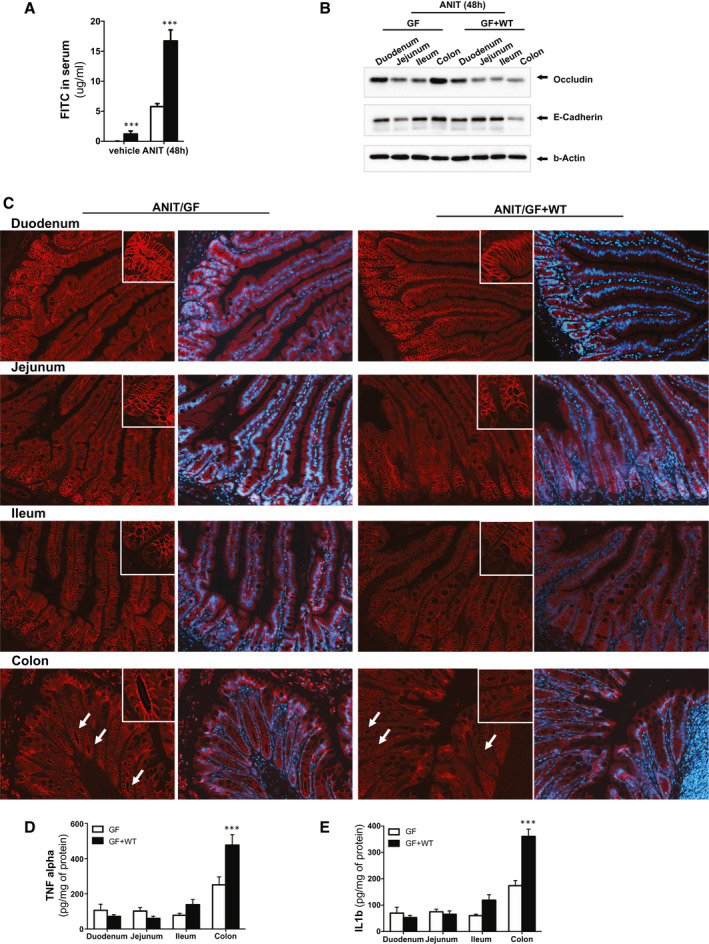
The intestinal microbiome exacerbates intestinal permeability during ANIT‐induced cholestasis. (A) Quantification of circulating FITC in serum samples from GF and GF + WT mice after vehicle and ANIT (48 hours). (B) Western blotting analysis on intestinal protein extracts from duodenum, jejunum, ileum, and colon showing reduced expression of tight junctions in GF + WT mice particularly pronounced in the colon. (C) Immunofluorescence staining on intestinal sections supporting reduced apical occludin expression in ANIT/GF + WT mice. (D) TNFα and (E) IL1β protein expression determined by ELISA on protein extracts isolated from duodenum, jejunum, ileum, and colon showing more pronounced inflammation in colons from ANIT/GF + WT mice. Values are mean ± SEM. *P < 0.05; **P < 0.01; ***P < 0.001 (vehicle/GF + WT vs. ANIT/GF + WT). Abbreviation: ELISA, enzyme‐linked immunosorbent assay.

Our results demonstrate that cholestasis is sufficient to moderately increase intestinal permeability in sterile mice given that ANIT/GF mice showed increased fluorescein isothiocyanate (FITC) presence in serum after oral administration (Fig. 5A). Also, conventionalization with a WT microbiome was sufficient to moderately increase intestinal permeability in vehicle/GF + WT mice, which was further exacerbated in response to ANIT‐induced cholestasis, as evidenced by >10‐fold increase in FITC presence in serum (Fig. 5A), supporting the implication of the microbiome in contributing to intestinal permeability.

We further assessed the intestinal permeability specifically in each part of the small intestine and colon. We found that expression of TJ proteins was primarily reduced in the colon from ANIT/GF + WT mice, supporting that this area of the intestine is the most permeable during cholestasis (Fig. 5B). These results were confirmed by immunofluorescence staining for occludin on intestinal sections that showed reduced apical membrane staining and cytoplasmic translocation in colonic enterocytes of ANIT/GF + WT mice compared to ANIT/GF (Fig. 5C).

In accordance with the increased intestinal permeability after ANIT treatment, we found elevated circulating LPS binding protein (LBP) in ANIT/GF + WT mice serum samples compared to vehicle/GF + WT animals (Supporting Fig. S3A).

Intestinal TJ protein expression is tightly regulated by inflammation.^(^ ^29^ ^)^ Analysis of individual regions of the intestine in GF + WT mice after ANIT treatment showed low TNF and IL1β expression levels in the duodenum and jejunum that were mildly increased in the ileum and reached the highest concentrations in the colon (Fig. 5D, E). TNF and IL1β expression was moderately up‐regulated in ANIT/GF mice, associated with a mild attenuation in TJ protein expression (Supporting Fig. S4A), suggesting regulatory crosstalk between bile acids and the immune system to preserve intestinal barrier function independently of the microbiome.

These observations were confirmed in 0.1% DDC–treated GF and GF + WT mice, which showed mildly decreased expression of TJ proteins and increased inflammation in the ileum, effects which were most pronounced in the colon of GF + WT mice (Supporting Fig. S5A‐C).

Overall, our results support that cholestasis contributes to increased intestinal inflammation and permeability that is further exacerbated in the presence of the microbiome.

### ANIT‐Induced Cholestasis Promotes Changes in the Microbiome Composition of GF + WT Mice

**Fig. 6:**
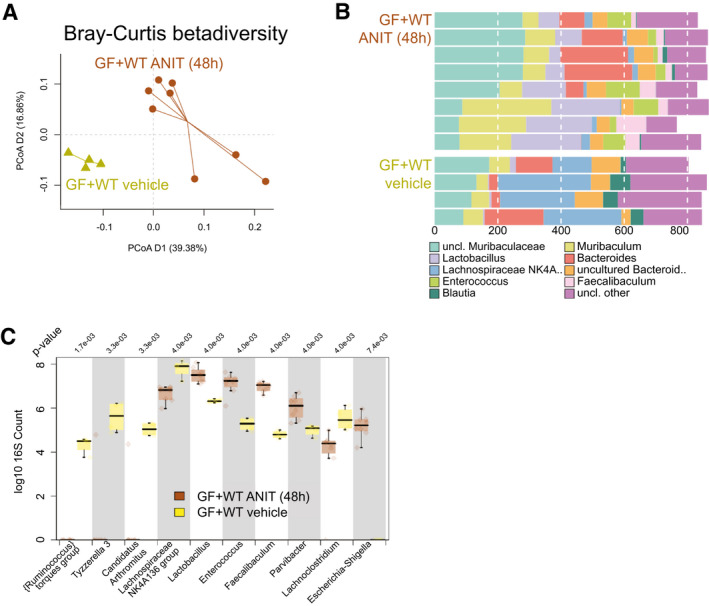
Intestinal microbiome composition changes during ANIT‐induced cholestasis. (A) Genus level PCoA (Bray‐Curtis distance) after 16s rRNA sequencing of intestinal microbiome composition. (B) Composition plot of the 10 most abundant taxonomic groups at genus level. Note that because of the often‐incomplete taxonomy, in some cases either the next known taxonomic level was chosen to represent the OTUs, whereas “Lachnospiraceae NK4A 136 group” does refer to a taxonomic group within family Lachnospiraceae. (C) Box plot of genera most significantly different between vehicle/GF + WT (n = 4) and ANIT/GF + WT (n = 8) mice. *P* value is shown above the plot; after multiple testing, all shown taxa were significantly different. *P < 0.05; **P < 0.01; ***P < 0.001 (vehicle/GF+WT vs. ANIT/GF + WT). Abbreviations: OTUs, operational taxonomic units; PCoA, principal coordinates analysis; uncl., unclassified.

Previous studies have shown that changes in the microbiome are associated with altered TJ protein expression.^(^ ^30^, ^31^ ^)^ We performed 16s ribosomal RNA (rRNA) sequence analysis of fecal samples with LotuS^(^ ^32^ ^)^ and found significant taxonomical changes in the intestinal microbiome during ANIT‐induced cholestasis in GF + WT mice, after correcting for absolute bacteria counts (Fig. 6A). Composition at genus level was significantly different in vehicle/GF + WT compared to ANIT/GF + WT (*P* = 0.002 per multivariate analysis of variance [MANOVA]; Fig. 6B,C). Univariate analysis showed an increased presence of *Enterococcus*, *Lactobacillus*, and *Escherichia* in ANIT/GF + WT when compared to vehicle/GF + WT (Fig. 6C), in line with findings during cholestasis in PSC and PBC patients.^(^ ^12^, ^13^, ^14^, ^15^, ^16^ ^)^

Overall, our results show that the increased intestinal permeability and inflammation observed during cholestasis correlated with changes in the microbiome composition.

### Macrophage Activation by the Inflammasome Mediates the Detrimental Impact of the Intestinal Microbiome in Contributing to Cholestatic‐Induced Liver Injury

To determine the contribution of macrophages to liver injury during cholestasis in conventionalized (GF + WT) mice, we depleted macrophages with clodronate‐loaded liposomes. In parallel, to further dissect the mechanisms underlying macrophage activation during cholestasis, we inhibited the inflammasome using the inhibitor, MCC950, that specifically targets Nlrp3.^(^ ^33^ ^)^

**Fig. 7:**
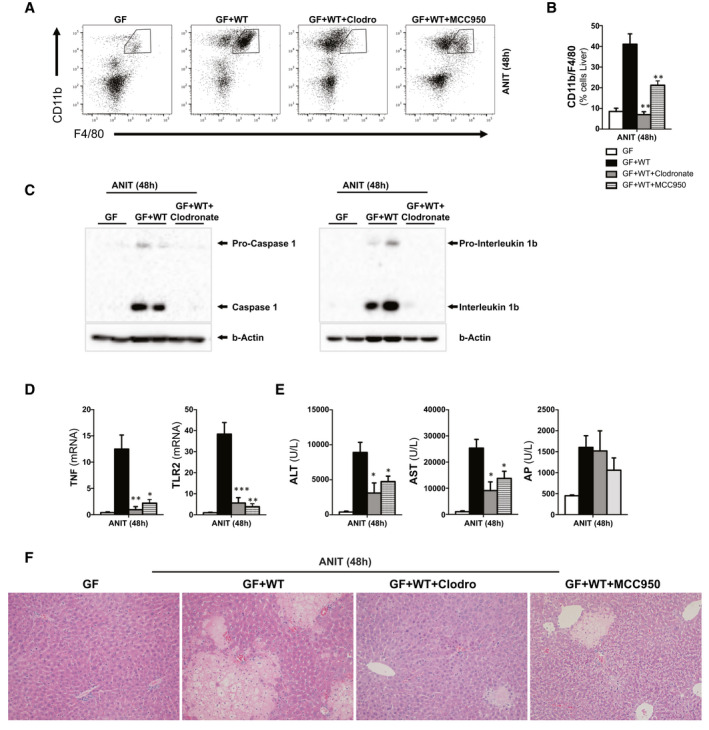
Depletion of macrophages and specific inhibition of Nrlp3 protects the liver from cholestasis‐induced liver injury. (A) FC analysis on liver‐isolated immune cells from GF, GF + WT, clodronate/GF+WT, and MCC950/GF + WT mice all treated with ANIT for 48 hours to induce cholestasis and further (B) quantification showing reduced macrophage infiltration in clodronate‐ and MCC950‐treated mice. (C) Western blotting analysis of whole‐liver lysates showing whole and cleaved caspase 1 and whole and cleaved IL1B. (D) qPCR analysis on liver extracts showing decreased inflammation, (E) reduced transaminase levels, and AP and (F) improved liver parenchyma status in H&E staining. n = 4‐8 animals per treatment group were analyzed. Values are mean ± SEM. *P < 0.05; **P < 0.01; ***P < 0.001 (ANIT/GF + WT vs. clodronate/GF + WT and MCC950//GF + WT). Abbreviations: ALT, alanine aminotransferase; AST, aspartate aminotransferase; FC, flow cytometry; H&E, hematoxylin and eosin; TLR2, Toll‐like receptor 2.

Our results show that depletion of macrophages (Fig. 7A,B) correlated with a significant attenuation of inflammasome activation given that both cleaved caspase 1 and cleaved IL1β protein expression was practically undetectable in clodronate‐treated conventionalized GF + WT mice after ANIT (Fig. 7C). This finding was in line with the reduced inflammation in ANIT/GF + WT mice after clodronate (Fig. 7D). Likewise, the specific inhibition of the inflammasome activation by Nrlp3 (Supporting Fig. S6A,B) also reduced the infiltration of macrophages in the liver and attenuated inflammation in MCC950‐treated ANIT/GF + WT mice (Fig. 7A,B,D).

Ultimately, both the inhibition of macrophages and of Nrlp3 markedly attenuated liver injury in conventionalized GF + WT mice after ANIT treatment, as evidenced by significant reduction of serum transaminases (Fig. 7E) and improved liver histology (Fig. 7F). The comparable AP levels in clodronate and MCC950/GF + WT mice (Fig. 7E) to those found in ANIT/GF + WT mice, together with the increased accumulation of bile acids in the liver and the comparable presence in serum and fecal samples (Supporting Fig. S7A‐C; Supporting Tables S7‐S9), supported that the protection exerted by macrophage/inflammasome depletion was not resulting from a lower degree of cholestasis.

### Activation of Macrophages by the Inflammasome Contributes to Intestinal Permeability and Influences the Microbiome Composition During Cholestasis in GF + WT Mice

The protection observed in macrophage‐ and inflammasome‐depleted mice could be a result of the reduced liver injury or of intestinal‐dependent events, including a lower permeability that would limit the translocation of bacteria from a leaky intestine into the liver.

**Fig. 8:**
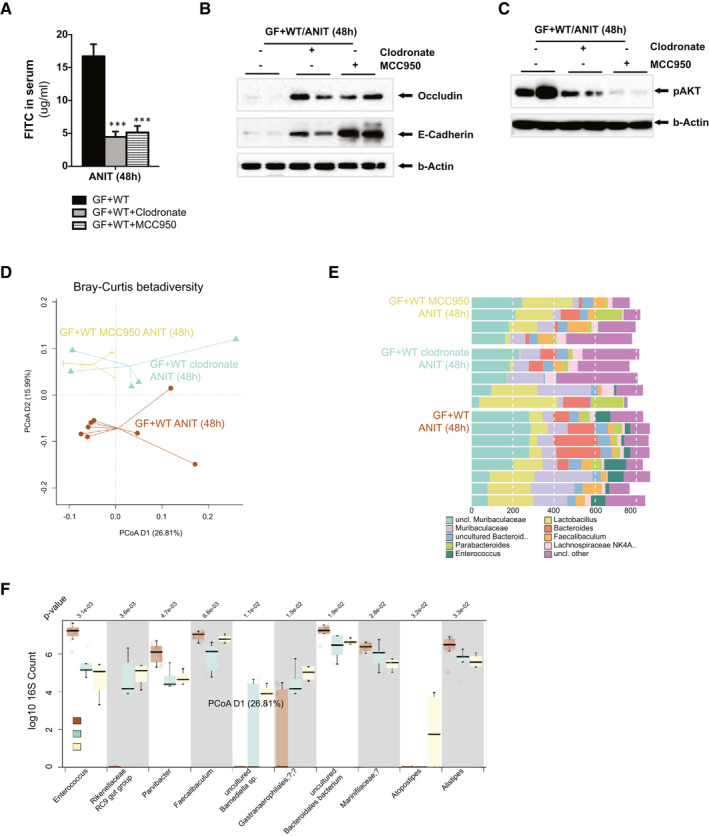
Macrophage depletion and inflammasome inhibition promote changes in the intestinal microbiome composition and reduce intestinal permeability during ANIT‐induced cholestasis. (A) Quantification of circulating FITC in serum samples from GF + WT, clodronate/GF + WT, and MCC950/GF + WT mice after ANIT (48 hours). (B) Western blotting analysis on colon protein extracts showing increased expression of TJs associated with (C) reduced phosphorylation of AKT in clodronate/GF + WT and MCC950/GF + WT mice. (D) Genus level PCoA (Bray‐Curtis distance) after 16s rRNA sequencing of intestinal microbiome composition. (E) Composition plot of the 10 most abundant genera. (F) Box plot of genera most significantly different between ANIT/GF + WT (n = 8) and clodronate/GF+WT (n = 5) and MCC950/GF+WT (n = 4) mice. *P* value is shown above the plot, after multiple testing; all shown taxa were significantly different. n = 4‐8 animals per treatment group were analyzed. Values are mean ± SEM. ***P < 0.001 (ANIT/GF + WT vs. clodronate/GF + WT and MCC950//GF + WT). Abbreviations: PCoA, principal coordinates analysis; uncl., unclassified.

Accordingly, with the role of inflammation in controlling TJ expression, we found a reduction of intestinal permeability in clodronate/GF + WT mice and MCC950/GF + WT mice during ANIT‐induced cholestasis, evidenced by lower detection of FITC in the circulation (Fig. 8A). In addition, we found restoration of intestinal TJ in macrophage‐ and inflammasome‐depleted GF + WT mice despite ANIT treatment, as shown by increased colonic occludin and E‐cadherin protein expression (Fig. 8B). The marked reduction of intestinal permeability resulting from macrophage and inflammasome depletion was further supported by the lower circulating LBP in clodronate‐ and MCC950‐ANIT/GF + WT mice when compared to ANIT/GF + WT animals (Supporting Fig. S8A).

Several mechanisms have been proposed to mediate the regulation of TJ by immune response, including activation of the phosphoinositide 3‐kinase (PI3K)/protein kinase B (AKT) pathway.^(^ ^34^, ^35^ ^)^ We found strong AKT phosphorylation in the colon of ANIT/GF + WT mice whereas inhibition of macrophages and the inflammasome significantly attenuated AKT phosphorylation (Fig 8C), implicating this pathway in mediating the inflammation‐TJ cross‐regulation.

Analysis of the fecal taxonomic composition evidenced that inhibition of both the macrophages and of the inflammasome in ANIT‐treated mice led to significant changes in microbiome diversity when compared to ANIT/GF + WT mice (*P* = 0.044 and *P* = 0.018 per MANOVA, respectively; Fig. 8D,E). No significant compositional differences were found between clodronate‐ANIT/GF + WT and MCC950‐ANIT/GF + WT groups (*P* = 0.55), demonstrating that inhibition of the inflammasome had a comparable effect to the depletion of macrophages on modulating intestinal microbiome composition.

Further analysis of microbiome composition at the genus level showed that during ANIT‐induced cholestasis, MCC950 treatment reduced the presence of *Enterococcus* and *Parvibacter* and increased the abundance of uncultured *Barnesiella* sp. and *Rikenellaceae RC9*, as compared to ANIT/GF + WT samples (Fig. 8F). In clodronate‐ANIT/GF + WT mice, we observed similar trends to MCC950‐treated animals (Fig. 8F), in paired comparisons however, no significance could be shown after multiple testing correction.

Overall, our results show that macrophages regulate intestinal permeability and microbiome composition during cholestasis through the inflammasome, and that this contributes to cholestatic disease progression by synergizing with bile acids to mediate hepatocellular injury in the liver (Table 1).

**Table 1:**
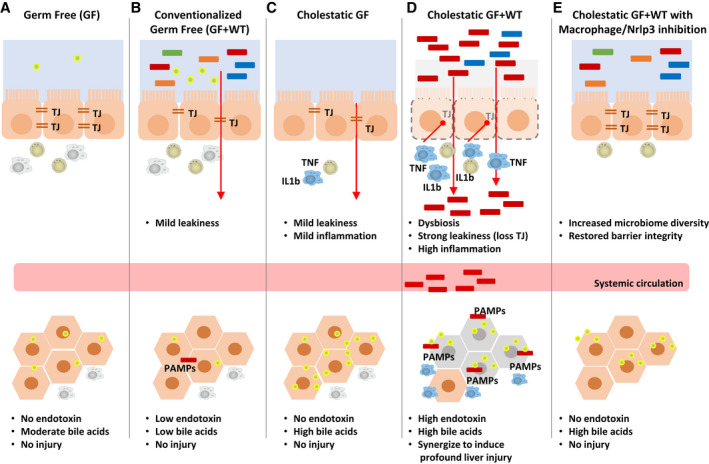
The Intestinal Microbiome Contributes to Liver Injury During Cholestasis by Increasing Intestinal Permeability Through Macrophage‐Inflammasome Activation

(A) GF mice present increased liver bile acids with no injury associated. (B) Conventionalization of GF mice leads to mild intestinal permeability. (C) Induction of cholestasis in GF mice leads to mild intestinal leakiness and inflammation that has no damaging impact on the liver, whereas (D) cholestasis in GF + WT mice caused significant intestinal permeability and inflammation and liver injury where endotoxin synergizes with bile acids to promote cell death. (E) Depletion of macrophages and Nrlp3 restores intestinal TJ protein expression and reduces liver injury associated with lower leakiness of intestinal endotoxin.

Abbreviation: PAMPs, pathogen‐associated molecular patterns.

## Discussion

In this study, we demonstrate that the intestinal microbiome contributes to sensitize hepatocytes to bile‐acid–induced cell death during cholestasis. In addition, we provide mechanistic evidence of the key role of activation of the inflammasome in macrophages as a contributor of cholestatic liver injury by regulating intestinal permeability and intestinal microbiome composition.

Human cholestatic disease associates with changes in microbiome composition,^(^ ^12^, ^13^, ^14^, ^15^, ^16^ ^)^ though whether the intestinal microbiome actively contributes to cholestatic disease or is a mere bystander of progression of the disease remains largely undefined.

The few mechanistic studies performed in mice investigating the contribution of the microbiome to the pathogenesis of cholestatic disease have provided conflicting results. Whereas some work supports the beneficial role of the microbiome in attenuating the progression of cholestasis and fibrosis,^(^ ^36^, ^37^ ^)^ recent studies suggest that the microbiome contributes to liver injury during cholestasis.^(^ ^37^, ^38^ ^)^ These controversial findings support the need for further research to establish the role of the microbiome in the pathogenesis of human cholestatic disease.

Although murine experimental models may not fully recapitulate human disease, ANIT and feeding with 0.1% DDC are well‐accepted models of sclerosing cholangitis relevant to PSC.^(^ ^40^, ^41^ ^)^ Moreover, the impact on intestinal permeability and microbiome composition observed in these preclinical models support their suitability to unravel the mechanisms underpinning the pathogenesis in PSC and PBC.^(^ ^12^, ^13^, ^14^, ^15^, ^16^, ^17^, ^18^ ^)^

In our present study, we establish that intestinal‐derived endotoxin is required for the cytotoxic action of bile acids in hepatocytes, given that their accumulation in the liver cannot trigger liver injury *per se* in a sterile model system, specifically GF mice. Significant resistance of GF mice to cholestatic‐induced liver injury could arguably result from the differential bile acid metabolism and/or pool composition (dominated by hydrophilic species), resulting from the lack of a microbiome. Analysis of xenobiotic and bile acid metabolism in the liver pointed to a decreased capacity to detoxify and export bile acids in GF mice. Nevertheless, this had no significant impact on the overall cholestasis after ANIT and 0.1% DDC treatments, supporting that the protection observed in GF mice was not resulting from a different bile acid metabolism.

In GF mice, the bile acid pool is dominated by more hydrophilic, less toxic, species (Supporting Tables S1‐S6). To rule out that the protection from cholestasis‐mediated liver injury observed in sterile mice was attributed to different bile acid composition, we performed *in vitro* studies where we exposed primary hepatocytes isolated from GF and GF + WT to bile acids. We confirmed that, in our experimental setting, cultured hepatocytes retained key bile acid metabolic characteristics,^(^ ^42^ ^)^ including the capacity to hydroxylate bile acids. Thus, whereas GF hepatocytes showed increased α/β‐MCA levels when exposed to CDCA, the conversion of DCA into CA was comparable in GF and GF + WT cells. Exposure to CDCA and DCA caused profuse caspase 3 activity and cell death in GF + WT hepatocytes compared to GF cells, suggesting that protection from bile‐acid–induced cell death found in GF mice was not only resulting from the increased hydroxylation of CDCA into MCA species. Likewise, stimulation with GCA also caused significantly more cell death in GF + WT hepatocytes compared to GF cells whereas TCA had only a mild impact on cell death in cultures. Importantly, pretreating GF hepatocytes with LPS significantly increased bile‐acid–mediated hepatocyte cell death, which seemed independent from the increased capacity of GF hepatocytes to hydroxylate CDCA into α‐MCA.

Overall, our results support that endotoxin and bile acids act synergistically to promote hepatocellular cell death during cholestatic liver disease. Based on the known physiological low‐grade leakage of bacterial products from the intestine,^(^ ^43^ ^)^ it is tempting to speculate that this circulating endotoxin (pre)sensitizes hepatocytes to bile‐acid–induced cell death when cholestasis occurs.

Intestinal permeability is regulated by TJ proteins, whose expression and cellular localization can be modulated by inflammation.^(^ ^29^ ^)^ We therefore investigated whether macrophage‐mediated inflammation may influence intestinal permeability in the context of cholestatic liver disease. Accordingly, we demonstrate that during cholestasis, elevated intestinal inflammation associates with a reduction in TJ protein expression and increased permeability, particularly in the colon. Our further mechanistic studies showed that macrophages, through activation of the inflammasome, play a key role in the regulation of intestinal permeability, given that their depletion led to the restoration of TJ protein expression in the colon and the significant reduction in leakage of bacterial products into the circulation.

A recent study highlighted that administration of a pan‐caspase inhibitor (inhibiting caspases 3, 6, and 8) improved the liver phenotype in cholestatic mice by reducing intestinal permeability.^(^ ^38^ ^)^ The role of intestinal Nrlp3 in mediating these effects was proposed in that study, although the impact of the specific inhibition of Nrlp3 was not tested. Importantly, by using a specific inhibitor, we here provide evidence that Nrlp3 activation is key to the control of intestinal permeability by controlling TJ protein expression. Our results suggest that activation of AKT may mediate the regulation of TJ expression by macrophages, supporting the previously described role of PI3K/signaling in mediating the effects of TNF in down‐regulating TJ expression described in human colonic monolayers.^(^ ^34^, ^35^ ^)^

The role of the microbiome in educating the intestinal immune system to generate a tolerant environment has been widely studied.^(^ ^1^ ^)^ Likewise, the influence of the immune system in regulating intestinal defense against pathogens while remaining tolerant to the resident microbiome is established. Nonetheless, the specific mechanisms and signaling pathways involved in regulating the influence of the immune system in the intestinal microbiome composition remain largely undefined. There is evidence that during aging, increased intestinal permeability and systemic low‐grade inflammation (inflammaging) are associated with decreased microbial diversity.^(^ ^4^, ^44^ ^)^ However, aged TNF‐knockout mice have comparable microbiome composition and intestinal barrier function to young mice,^(^ ^4^ ^)^ supporting a causative relationship between TNF‐driven inflammation, changes in the microbiome, and intestinal permeability. In zebrafish, Early et al. showed that a specific subset of intestinal macrophages is required to ensure the colonization of gut microbes and thus determine microbiome composition.^(^ ^45^ ^)^ Our results show that modulating inflammation by inhibition of macrophages/inflammasome affects microbiome composition during cholestasis, which may further contribute to promote intestinal permeability, thus aggravating cholestatic liver disease by allowing the translocation of bacterial products. Our observations in GF mice show that colonization with a WT microbiome leads to a moderate reduction in TJ expression, supporting the influence of the microbiome in regulating intestinal permeability. Our results are in agreement with previous studies where administration of (low doses of) bacterial LPS^(^ ^46^ ^)^ or presence of pathogenic bacteria (*Escherichia coli*)^(^ ^47^ ^)^ led to the disruption of TJ, possibly mediated by inflammation (TNF).

Our results also show that cholestasis induced an increase in inflammation independently of the presence of the microbiome in ANIT/GF mice that correlated with mild attenuation of the expression of TJ in the colon. These results point to a role of bile acids in preserving intestinal immune cell homeostasis and barrier integrity during health. Thus, the absence of bile acids in the intestine during cholestasis may modulate intestinal immune cell activation and hence increase permeability at early stages of the disease, allowing the translocation of bacteria (and their products) into the liver, initiating injury in synergy with bile acids. The persistent decrease of bile acid flux into the intestine at later stages may lead to profound changes in microbiome composition and to increased inflammation, which may further contribute to reducing TJ expression and increasing intestinal permeability.

Our present work points to preserving intestinal barrier function, by mechanisms including the regulation of (1) intestinal macrophages through the inflammasome and (2) the composition of the microbiome, as therapeutic approaches to treat cholestatic liver disease. To define the functionality of the microbiome associated with liver disease and to better understand how this influences intestinal inflammation and barrier function will be essential to develop microbial therapeutics to treat or prevent cholestatic disease. There is increasing evidence of the efficacy of changing microbiome composition by the supplementation of liver bacteria and/or prebiotics on preserving intestinal barrier integrity and reducing liver injury in the context of alcoholic and nonalcoholic steatohepatitis.^(^ ^48^, ^49^ ^)^ Thus, future studies will determine the impact of interventions aimed at changing microbiome composition during cholestatic liver disease progression.

In summary, we here provide insights into the synergistic effects of bile acids and bacterial (components) in triggering hepatocellular cell death in the context of cholestasis. Also, we propose that activation of intestinal macrophages, through the inflammasome, promotes intestinal permeability and influences microbiome composition, permitting the leakage of bacterial products to the liver where they contribute to hepatocellular cell death. Overall, our study points to the regulation of intestinal macrophage/inflammasome‐microbiome axis as a potential target for therapeutics to treat this condition.

## Author Contributions

A.I.‐T. and M.E. contributed equally to this work. A.I.‐T., M.E., M.M.‐G., A.M.P., A.P., M.G. and N.B. performed experiments, sample analysis and data collection. A.I.‐T., M.E., M.M.‐G. and N.B. performed data analysis. A.B. and A.G. provided with germ free mice and performed experiments. M.P. performed MS‐HPLC, data collection and data analysis. D.B. performed sample preparation and 16s rRNA sequencing. A.P., S.M.R. and F.H. critically revised the manuscript. F.H. did sequencing data analysis, bioinformatic data analysis and interpretation. N.B. and F.H. prepared figures. S.M.R. and N.B. obtained funding. N.B. generated study concept, experimental design, interpreted data and applied statistical analyses, did study and staff supervision and wrote manuscript. All authors read and approved the final version of the manuscript.

## Supporting information

Supplementary Material
