# Supplementary material for: Intestinal Microbiome‐Macrophage Crosstalk Contributes to Cholestatic Liver Disease by Promoting Intestinal Permeability in Mice
Source: Hepatology. 2020 Dec 23;72(6):2090–108. doi: 10.1002/hep.31228 (PMC7839474; doi:10.1002/hep.31228)
Supplement: Supplementary file 1 — Supplementary Material [file HEP-72-2090-s001.pdf]

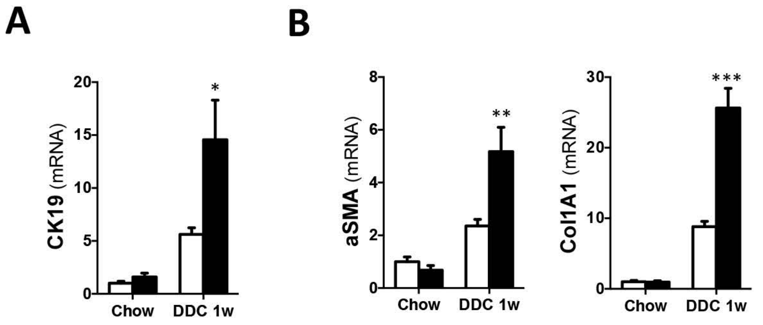

Supplemental Figure 1

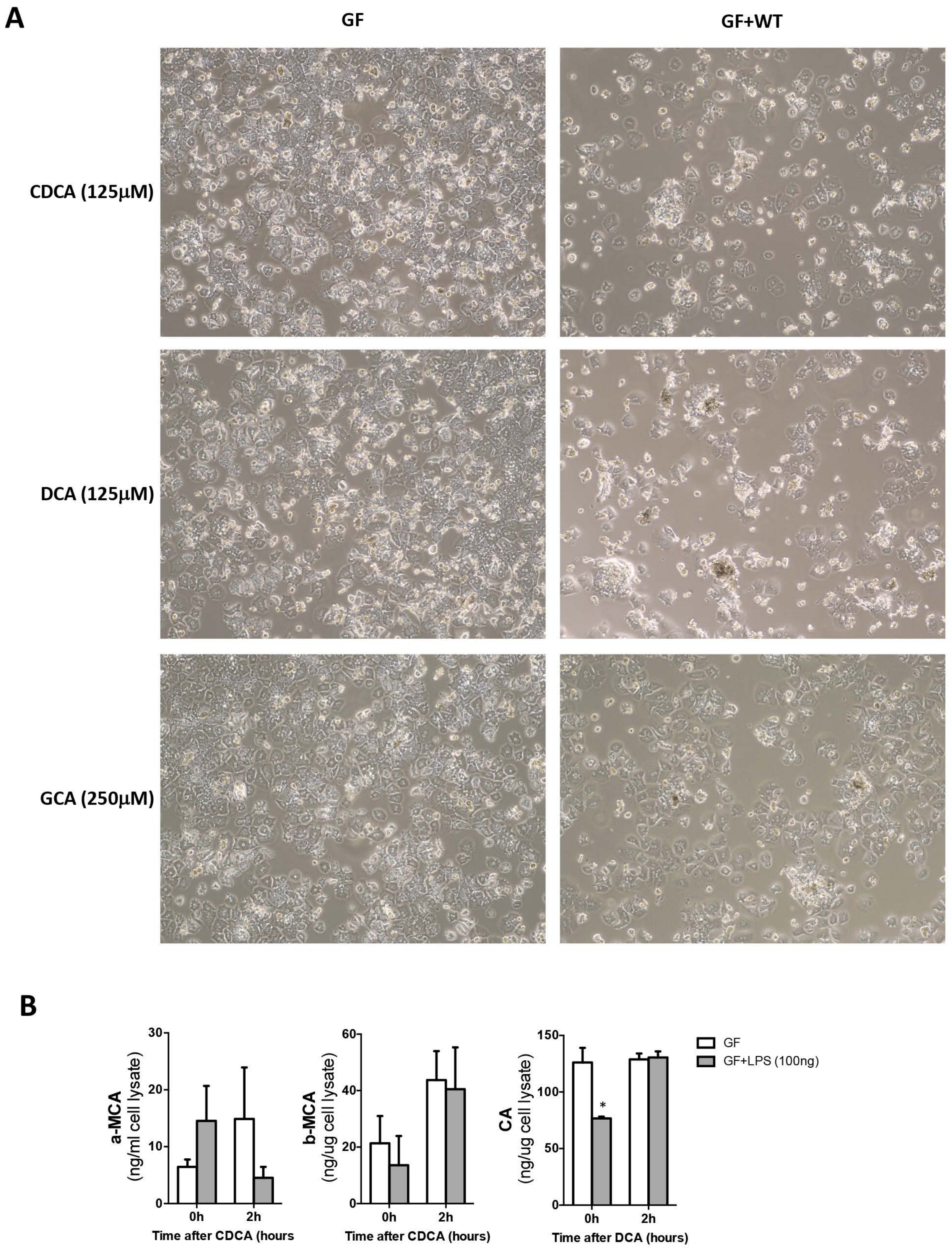

Supplemental Figure 2

A

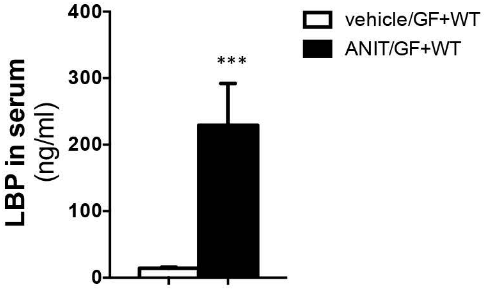

Supplemental Figure 3

A

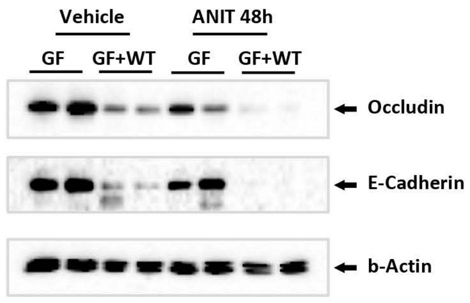

Supplemental Figure 4

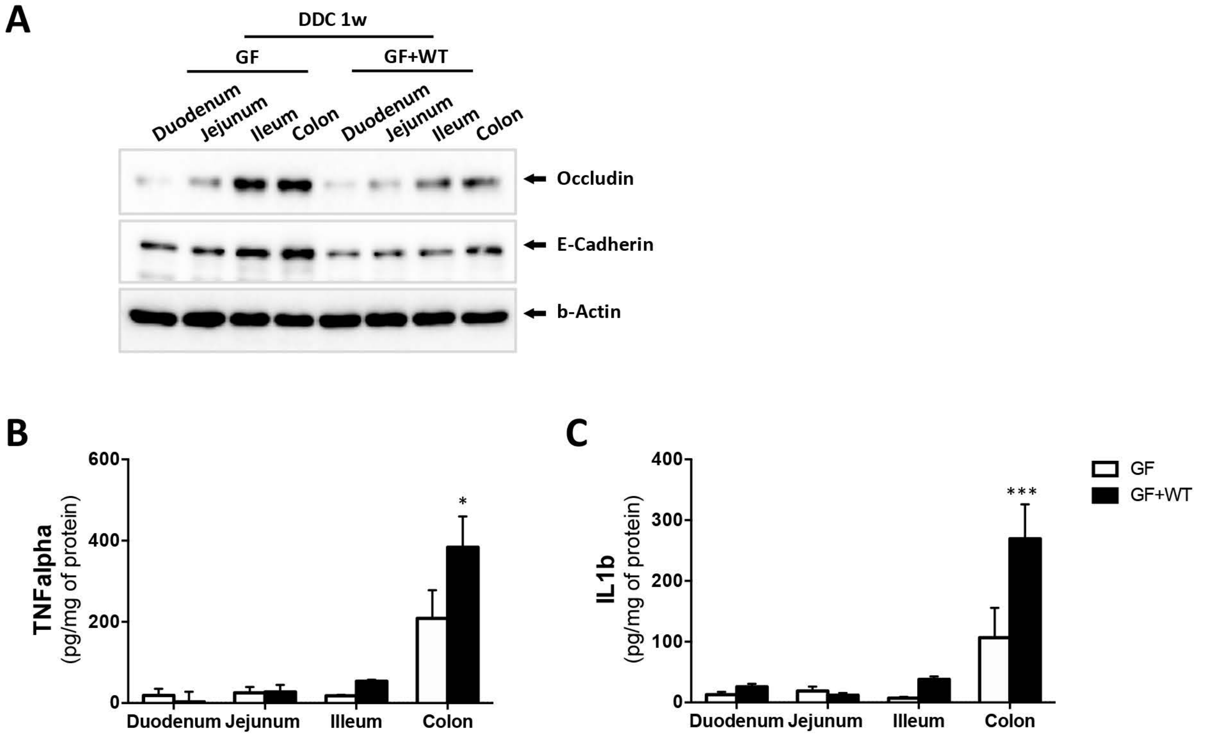
A
DDC 1w
GF
GF+WT
Duodenum
Jejunum
Ileum
Colon
Duodenum
Jejunum
Ileum
Colon
Occludin
E-Cadherin
b-Actin
B
TNFalpha
(pg/mg of protein)
600
400
200
0
*
Duodenum
Jejunum
Illeum
Colon
C
IL1b
(pg/mg of protein)
400
300
200
100
0
***
Duodenum
Jejunum
Illeum
Colon
GF
GF+WT

Figure 5

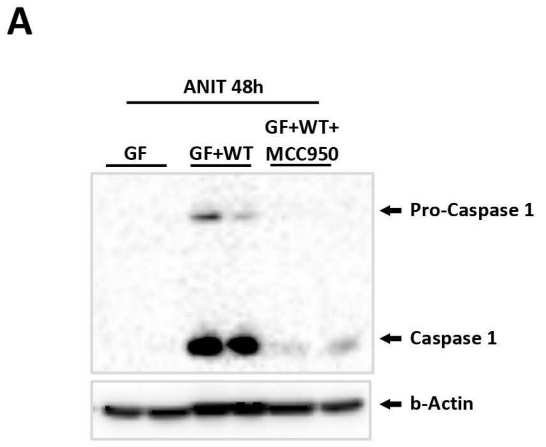

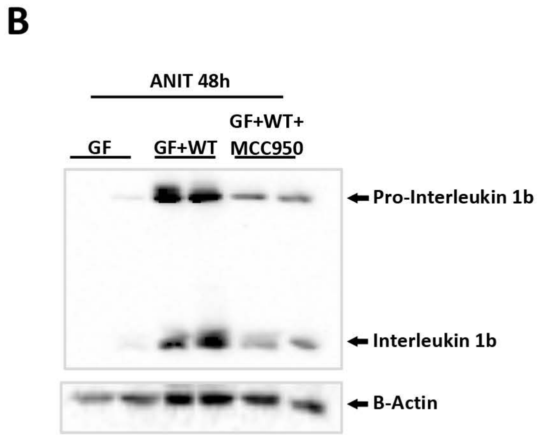

Supplemental Figure 6

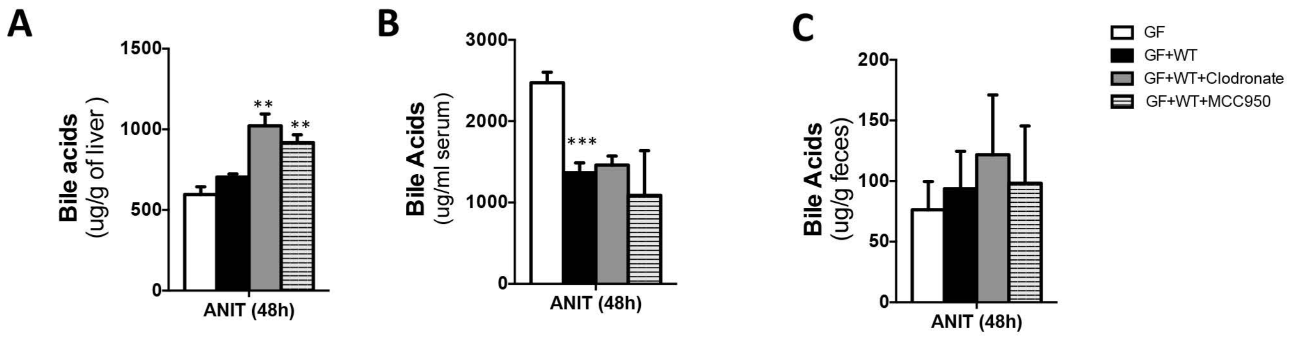

Supplemental Figure 7

A

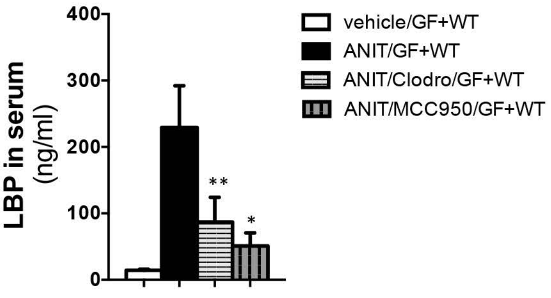

Supplemental Figure 8

Supplemental Table 1. Bile acids in liver (μg/g)

	Vehicle		ANIT (48h)	
	GF	GF+WT	GF	GF+WT
**CA**	0.19±0.07	0.23±0.13	0.45±0.26	0.64±0.20
**CDCA**	0.07±0.01	0.06±0.03	0.05±0.01	0.08±0.00**
**a-MCA**	0.04±0.01	0.12±0.09*	0.22±0.26	1.00±0.08***
**b-MCA**	2.82±1.56	0.39±0.20*	79.92±39.09	13.05±2.38**
**T-a-MCA**	8.11±7.55	13.24±8.35	10.01±6.43	63.72±14.27***
**T-b-MCA**	218.36±85.88	42.26±19.9*	337.38±83.53	315.70±14.18
**TCA**	74.33±39.11	45.14±27.11	150±26.12	296.27±21.42***
**TDCA**	0.05±0.05	2.69±1.76**	0.03±0.01	3.41±1.21***
**TUDCA**	5.64±4.95	1.52±0.69	15.60±7.16	2.69±2.39**
**TCDCA**	2.67±2.42	1.45±0.75	1.98±2.34	1.02±0.42
**GCA**	0.10±0.03	0.22±0.07*	0.14±0.023	1.03±0.25***
**DCA**	0.20±0.04	0.21±0.13	0.16±0.06	0.53±0.65

Supplemental Table 2. Bile acids in serum (μg/ml)

	Vehicle		ANIT (48h)	
	GF	GF+WT	GF	GF+WT
CA	0.04±0.02	0.13±0.09*	0.31±0.18	1.67±2.20
CDCA	0.03±0.02	0.03±0.02	0.03±0.02	0.01±0.01*
a-MCA	0.01±0.01	0.05±0.03*	0.00±0.00	1.94±1.04***
b-MCA	0.07±0.08	0.08±0.03	74.86±57.46	15.41±4.68*
T-a-MCA	0.07±0.16	0.09±0.17	4.19±11.85	35.19±30.24*
T-b-MCA	3.58±6.00	0.44±0.25	1605.76±192.05	302.21±100.33***
TCA	1.08±1.89	0.96±0.32	922.64±283.50	1003.87±212.10
TDCA	0.02±0.03	0.02±0.01	1.06±1.27	0.55±0.22
THDCA	0.05±0.07	0.02±0.00	9.50±2.98	0.77±0.52
GCA	0.06±0.05	0.14±0.03*	0.16±0.10	1.12±0.40***
DCA	0.02±0.01	0.12±0.12	0.02±0.01	0.01±0.01

Supplemental Table 3. Bile acids in faeces (μg/g)

	Vehicle		ANIT (48h)	
	GF	GF+WT	GF	GF+WT
CA	0.25±0.24	19.05±24.99	0.74±0.44	13.07±11.04*
CDCA	0.20±0.18	0.86±0.69*	0.31±0.21	1.23±2.83*
a-MCA	0.04±0.08	58.96±14.42***	0.01±0.02	4.75±2.70**
b-MCA	0.23±0.25	50.28±27.38**	9.19±6.60	9.32±3.50
T-b-MCA	85.82±44.51	28.98±49.56	57.47±36.15	0.82±1.29***
TCA	21.93±7.67	31.61±55.11	16.02±7.93	2.27±5.23***
TDCA	0.56±0.33	1.96±2.55	0.32±0.20	0.57±1.64
TCDCA	0.87±0.93	1.57±2.13	0.37±0.48	0.25±0.44
GCA	0.78±0.88	0.33±0.20	0.75±0.54	1.93±3.97
DCA	0.21±0.18	68.47±13.09***	0.57±0.32	41.31±52.20
LCA	0.20±0.14	3.29±2.36***	0.11±0.06	3.29±2.36*

Supplemental Table 4. Bile acids in liver (μg/g)

	Chow		DDC (1w)	
	GF	GF+WT	GF	GF+WT
**CA**	1.19±0.86	0.56±0.24	0.16±0.03	0.53±0.38
**CDCA**	0.09±0.05	0.01±0.01	0.09±0.02	0.10±0.02
**a-MCA**	0.69±0.41	0.37±0.30	0.05±0.01	0.41±0.21**
**b-MCA**	4.35±3.55	3.22±1.81	4.82±3.57	8.71±3.00
**T-a-MCA**	18.53±8.42	0.27±0.60	18.21±3.11	45.73±19.48*
**T-b-MCA**	83.79±8.42	24.37±13.07	339.52±62.07	279.72±54.10
**TCA**	56.25±15.62	74.2±37.48	120.33±11.24	122.14±44.29
**TDCA**	2.57±0.47	1.64±0.51	0.05±0.05	0.51±0.35*
**TUDCA**	2.42±0.52	0.94±0.38	6.09±0.62	3.15±1.00***
**TCDCA**	1.76±0.56	2.16±0.73	12.09±2.67	1.94±0.59***
**GCA**	0.29±0.07	0.14±0.04	0.18±0.10	0.35±0.31
**DCA**	0.40±0.34	0.1±0.13	0.28±0.03	0.26±0.09

Supplemental Table 5. Bile acids in serum (μg/ml)

	Chow		DDC (1w)	
	GF	GF+WT	GF	GF+WT
**CA**	0.03±0.02	0.06±0.07	0.02±0.02	2.80±2.63**
**CDCA**	0.02±0.02	0.02±0.02	0.01±0.01	0.01±0.02
**a-MCA**	0.03±0.02	0.01±0.01	0.01±0.03	0.98±0.66***
**b-MCA**	0.07±0.08	0.06±0.08	2.284±2.00	14.54±11.19**
**T-a-MCA**	0.05±0.07	0.02±0.03	6.06±5.49	11.23±7.83
**T-b-MCA**	3.72±4.53	0.03±0.04	627.92±312.00	142.43±57.18***
**TCA**	0.87±0.96	0.34±0.10	150.17±70.49	97.23±57.17
**TDCA**	0.00±0.01	0.01±0.01	2.07±0.85	3.83±1.42
**THDCA**	0.06±0.03	0.01±0.01	2.07±0.85	0.57±0.21***
**GCA**	0.09±0.06	0.09±0.05	0.07±0.05	0.19±0.08**
**DCA**	0.02±0.01	0.04±0.03	0.02±0.01	0.10±0.05**

Supplemental Table 6. Bile acids in faeces (μg/g)

	Chow		DDC (1w)	
	**GF**	**GF+WT**	**GF**	**GF+WT**
**CA**	0.05±0.05	2.47±1.50*	0.29±0.10	3.75±2.46*
**CDCA**	0.02±0.01	0.00±0.00*	0.18±0.04	0.22±0.05
**a-MCA**	0.02±0.02	47.64±7.17***	0.00±0.00	6.62±1.69**
**b-MCA**	0.19±0.29	36.36±15.83**	0.10±0.02	14.68±3.53***
**T-b-MCA**	136.27±36.10	2.51±1.20***	32.69±11.39	0.15±0.08***
**TCA**	36.30±8.92	6.35±3.12***	9.10±3.59	0.16±0.11***
**TDCA**	1.02±0.44	0.49±0.31	0.08±0.03	0.04±0.03*
**TCDCA**	0.02±0.01	0.61±0.35*	0.40±0.15	0.02±0.01***
**GCA**	0.07±0.08	0.15±0.10	0.19±0.05	0.13±0.07
**DCA**	0.04±0.03	184.9±62.05***	0.23±0.08	3.91±1.84**
**LCA**	0.13±0.12	10.36±2.17***	0.11±0.04	0.30±0.11**

Supplemental Table 7. Bile acids in liver (μg/g)

ANIT (48h)			
	**GF+WT**	**GF+WT+clodro**	**GF+WT+MCC950**
**CA**	0.64±0.20	1.43±0.20**	1.40±0.28**
**CDCA**	0.08±0.00	0.15±0.10	0.18±0.13
**a-MCA**	1.00±0.08	2.54±1.56	4.52±0.75*
**b-MCA**	13.05±2.38	42.99±20.61*	42.35±9.70**
**T-a-MCA**	63.72±14.27	100.64±63.49	104.87±14.74**
**T-b-MCA**	315.70±14.18	455.40±45.36***	370.05±57.82
**TCA**	296.27±21.42	392.49±100.69	367.66±19.55**
**TDCA**	3.41±1.21	1.63±2.11	4.59±5.09
**TUDCA**	2.69±2.39	11.92±1.28***	10.30±0.78***
**TCDCA**	1.02±0.42	3.03±1.28*	2.25±0.78*
**GCA**	1.03±0.25	0.77±0.53	1.09±0.39
**DCA**	0.53±0.65	0.32±0.12	0.36±0.14

Supplemental Table 8. Bile acids in serum (μg/ml)

ANIT (48h)			
	**GF+WT**	**GF+WT+clodro**	**GF+WT+MCC950**
**CA**	1.67±2.20	1.84±1.65	2.74±2.49
**CDCA**	0.01±0.01	0.02±0.00	0.01±0.01
**a-MCA**	1.94±1.04	1.58±1.26	4.97±0.48***
**b-MCA**	15.41±4.68	20.13±18.54	32.62±4.91**
**T-a-MCA**	35.19±30.24	26.38±20.75	19.978±30.25
**T-b-MCA**	302.21±100.33	410.38±132.28	272.12±59.20
**TCA**	1003.87±212.10	994.52±260.91	960.10±147.20
**TDCA**	0.55±0.22	0.90±0.35$^{p=0.05}$	0.90±0.19*
**THDCA**	0.77±0.52	1.52±1.33	3.12±0.91**
**GCA**	1.12±0.40	0.74±0.52	0.77±0.24
**DCA**	0.01±0.01	0.04±0.05	0.26±0.33

Supplemental Table 9. Bile acids in faeces (μg/g)

ANIT (48h)			
	**GF+WT**	**GF+WT+clodro**	**GF+WT+MCC950**
**CA**	13.07±11.04	5.06±2.80	3.73±1.65
**CDCA**	1.23±2.83	0.40±0.07	0.34±0.11
**a-MCA**	4.75±2.70	24.79±32.41*	20.79±23.99*
**b-MCA**	9.32±3.50	13.10±8.95	5.71±2.01
**T-b-MCA**	0.82±1.29	3.27±7.13	0.08±0.06
**TCA**	2.27±5.23	2.99±5.34	0.16±0.08
**TDCA**	0.57±1.64	0.10±0.09	0.05±0.03
**TCDCA**	0.25±0.44	0.08±0.07	0.01±0.02
**GCA**	1.93±3.97	0.26±0.13	0.19±0.07
**DCA**	41.31±52.20	60.78±62.75	55.42±54.06
**LCA**	3.29±2.36	5.10±5.73	4.96±7.08

Supplemental Material

Intestinal microbiome-macrophages crosstalk contributes to cholestatic liver disease by promoting intestinal permeability

Anna Isaacs-Ten[1]*, Marta Echeandia[1]*, Mar Moreno-Gonzalez[1], Arlaine Brion[2], Andrew Goldson[2], Mark Philo[2], Angela M Patterson[1], Aimee Parker[1], Mikel Galduroz[1], David Baker[3], Simon M Rushbrook[4], Falk Hildebrand[1,6], Naiara Beraza[1, 5, 7]

Supplemental Material and Methods

Materials

Chenodeoxycholic and deoxycholic acid (CDCA and DCA) were obtained from Sigma-Aldrich. Clodronate liposomes were obtained from Liposoma Research. MCC950, the specific inhibitor of Nrlp3, was obtained from Invivogen.

Bile acid determination

Bile acids were extracted from liver samples and analyzed using high performance liquid chromatography-tandem mass spectrometry we previously described (1). In brief, cleaned-up extracts were analysed using HPLC-mass spectrometry operated in multiple reaction monitoring (MRM) mode. Each sample (5 µl) was analysed using an Agilent 1260 binary HPLC coupled to an AB Sciex 4000 QTrap triple quadrupole mass spectrometer. HPLC was achieved using a binary gradient of solvent A (Water + 5mM Ammonium Ac + 0.012% Formic acid) and solvent B (Methanol + 5mM Ammonium Ac + 0.012% Formic acid) at a constant flow rate of 600 µl/min. Separation was made using a Supelco Ascentis Express C18 150 x 4.6, 2.7µm column maintained at 40°C. Injection was made at 50% B and held for 2 min, ramped to 95%B at 20 min and held until 24 minutes. The column equilibrated to initial conditions for 5 minutes.

The mass spectrometer was operated in electrospray negative mode with capillary voltage of -4500V at 550°C. Instrument specific gas flow rates were 25ml/min curtain gas,

GS1: 40 ml/min and GS2: 50 ml/min. Mass fragmentation was monitored and quantification was applied using Analyst 1.6.2 software to integrate detected peak areas relative to the deuterated internal standards.

Mouse hepatocyte isolation and culture

Primary hepatocytes were isolated from germ free (GF) and conventionalised GF mice GF+WT by perfusion of the liver and further digestion with collagenase I (Worthington). Cells were washed, pelleted and plated on rat collagen type I (BD Biosciences) pre-coated plates with Minimum Essential Medium as we described in (1).

Microscopic images of hepatocyte cultured were taken using EVOS XL Core imaging system (Thermofisher).

Histology, immunohistochemistry and immunofluorescence

Liver tissues were immediately fixed in 10% neutral formalin and embedded in paraffin. Tissue blocks were further sectioned, dewaxed and hydrated. Liver slides were stained with H&E for pathological analysis and fibrosis was determined with Sirius Red staining. In all cases, a representative picture of 5-10 fields per sample is shown.

Immunohistochemistry (IHC) on paraffin embedded sections was performed using an anti-CK19 (Developmental Studies Hybridoma Bank, University of Iowa) diluted in antibody diluent (Dako), after which an anti-Rat HRP labelled secondary antibody was applied. IHC was developed using the DAB^{+} chromogen system (Dako) and nuclei were counterstained with hematoxylin.

Immunofluorescence using an anti-Occludin antibody (AbCam) was used in paraffin cut sections of duodenum, jejunum, ileum and colon. A Cy3-labeled secondary antibody was used and slides were mounted in a Dapi-containing solution (VectorLabs).

RNA isolation and Quantitative Real-Time PCR

RNA was isolated from liver samples with QiAzol Reagent (Qiagen) followed by first

strand synthesis with random primers and reverse transcription using M-MLV Reverse Transcriptase (Invitrogen). Quantitative real-time PCR (qPCR) was performed using SYBR Green reagent (Life technologies) in a ViiA7 Real-time PCR detection system (Applied Biosystems). Gene expression was normalized using hypoxanthine phosphoribosyltransferase 1 (HPRT1) and TATA-box binding protein (TBP1) and expressed in times *versus* expression in control samples. Primer sequences can be provided under request.

Characterization of apoptotic hepatocyte death

Apoptotic cell death was determined by quantifying Caspase-3 activity primary hepatocytes using a fluorescence-labelled substrate following the manufacturer's instructions (Enzo) as we previously described (1).

Western Blot Analysis and ELISA

Proteins were extracted from snap frozen liver and intestinal tissues, using RIPA buffer containing 10 mM Tris-Cl (pH 8), 1 mM EDTA, 1% Triton X-100, 0.1% sodium deoxycholate, 0.1% SDS, 140 mM NaC and 1 mM PMSF.

Whole cell lysates were further resolved in sodium dodecyl sulphate-polyacrylamide gels and transferred to nitrocellulose membranes (Whatman) and transferred to nitrocellulose blotting membranes. Membranes were probed with Caspase 1, Interleukin 1β, Occludin primary antibodies (Santa Cruz biotechnologies) as well as E-Cadherin (AbCam). As a loading control, we used b-Actin (Sigma Aldrich) antibody. As secondary antibody, we used anti-mouse IgG–HRP-linked (Santa Cruz biotechnologies).

Determination of proinflammatory cytokines TNF and Interleukin 1β, expression was determined in intestinal protein extracts using R&D systems DuoSet.

Flow Cytometry

Immune cells were isolated from liver tissues after digestion with collagenase for 30 minutes at 37C. Samples were homogenised and passed through a mesh followed by successive washes and centrifuged for gradient separation using Percoll. Isolated immune cells were stained with CD45-APC-Cy7 (BD), CD11b-PE (BD) and F4/80-FITC (Myltenyi) antibodies. Flow cytometry analysis was performed using BD LSRFortessa and analysed using FlowJo software.

Intestinal permeability

Mice were gavaged with FITC-Dextran (44mg/100g) before sacrificed. FITC was detected in serum samples by fluorescence spectrophotometry (excitation:490nm, emission:530nm).

Bacterial genomic DNA isolation and 16s rRNA sequencing

Bacterial genomic DNA was isolated from faecal pellets using the MBP DNA Soil extraction kit. Genomic DNA was normalised to 5ng/µl with EB (10mM Tris-HCl) and libraries were performed.

Briefly, following a first PCR and clean-up using KAPA Pure Beads (Roche Catalogue No. 07983298001) a second PCR master mix was made up using P7 and P5 of Nextera XT Index Kit v2 index primers (Illumina Catalogue No. FC-131-2001 to 2004). Following the PCR reaction, the libraries were quantified using the Quant-iT dsDNA Assay Kit, high sensitivity kit (Catalogue No. 10164582) and run on a FLUOstar Optima plate reader. Libraries were pooled and run on a High Sensitivity D1000 ScreenTape (Agilent Catalogue No. 5067-5579) using the Agilent Tapestation 4200 to calculate the final library pool molarity. The pool was run on an Illumina MiSeq instrument using MiSeq® Reagent Kit v3 (600 cycle) (Illumina Catalogue FC-102-3003) following the Illumina recommended denaturation and loading recommendations which included a 20% PhiX spike in (PhiX Control v3 Illumina Catalogue FC-110-3001). The raw data was analysed locally on the MiSeq using MiSeq reporter.

16S sequence analysis

The LotuS 1.36 was used pipeline (2) in short amplicon mode with default quality filtering. Raw 16S rRNA gene reads were quality filtered to ensure a minimum length of 170 bp, not more than eight homonucleotides, no ambiguous bases, average quality >= 27 and an accumulated error below 0.5. For OTU clustering and denoising we used UPARSE (3), removing chimeric OTUs against the RDP reference database (http://drive5.com/uchime/rdp_gold.fa) with uchime merging reads with FLASH (4) and assigning a taxonomy using the LotuS LCA algorithms against Silva 128 reference database (5). We could assign on average 5500 ± 3108 reads to each sample that were of cyanobacterial origin. Further data analysis was conducted with R statistical language Version 3.00 (The R Foundation, https://www.r-project.org/) as described in Hildebrand et al. (6), employing the rtk software (7) or all data normalizations.

Statistical analysis

Data are expressed as mean ± standard error of the mean. Statistical significance was determined by two-way analysis of variance followed by a Student's t test. All data shown are representative of at less three independent experiments.

Supplemental Figure legends

Supplemental Figure 1 Germ Free mice show lower ductular reaction and fibrosis during cholestasis

(A) qPCR analysis on liver tissue showing increased CK19 and (B) fibrosis related genes in GF+WT mice after ANIT when compared to GF mice. Graphs are representative of n≥5 animals/treatment group (*P <0.05, **P <0.01, *** P <0.001 [GF vs GF+WT]).

Supplemental Figure 2 Bile acids induce more cell death in GF+WT cultured hepatocytes compared to GF cells

(A) Microscopical images showing that primary hepatocytes isolated from GF+WT mice were more sensitive to cell death than GF cells in response to (A) CDCA (125μM), DCA (125 μM), and GCA (250 μM). Pictures were taken using EVOS XL Core Cell Imaging System at 10x magnification. (B) MS-HPLC analysis of hepatocyte lysates to determine the presence of α/β-MCA and CA after CDCA and DCA treatment for 2 hours in hepatocytes pre-treated with LPS (100ng/ml) 4 hours before BA exposure.

Supplemental Figure 3 ANIT promotes increased circulation of bacterial products in GF+WT mice

(A) ELISA detecting LPS Binding Protein in serum samples showing increased circulation of LBP in ANIT-treated GF+WT mice compared to vehicle treated mice. Graph is representative of n≥5 animals/treatment group *** P <0.001 [vehicle/GF+WT vs ANIT/GF+WT]).

Supplemental Figure 4 The microbiome and ANIT treatment modulate expression of TJ proteins.

A) Western blot analysis on colonic protein extracts showing reduced expression of tight junctions proteins Occludin and ECadherin in GF+WT mice after colonisation and more

apparently after ANIT. Western blot shows samples representative of n≥5 animals/treatment group.

Supplemental Figure 5. 0.1%DDC promotes reduction of TJ protein expression and increase inflammation in the intestine

(A) Western blot analysis on intestinal protein extracts from duodenum, jejunum, ileum and colon showing reduced expression of ECadherin in the ileum of GF+WT mice that was more pronounced in the colon. (B) TNFα and (C) IL1β protein expression determined by ELISA on protein extracts isolated from the different parts of the intestine showing more pronounced inflammation in colons from 0.1%DDC/GF+WT mice. Values are mean ± SEM. *P <0.05, *** P <0.001 [0.1%DDC/GF *vs* 0.1%DDC/GF+WT]).

Supplemental Figure 6 MCC950 efficiently inhibits the activation of the inflammasome

(A) Western blot analysis of whole protein lysates from liver samples obtained from ANIT/GF, ANIT/GF+WT mice and ANIT/MCC950/GF+WT showing pro-Caspase 1 and cleaved Caspase and Pro-Interleukin 1 beta and cleaved interleukin 1 beta.

Supplemental Figure 7 Macrophage depletion and inflammasome inhibition does not alter the cholestatic phenotype observed after ANIT

Quantification of bile acid pool size in (A) livers, (B) serum and (C) fecal samples from ANIT/GF+WT vs ANIT/clodronate/GF+WT and ANIT/MCC/GF+WT mice by MS-HPLC. Graphs are representative of n=4-8 animals/treatment group.

Supplemental Figure 8 Macrophage depletion and inflammasome inhibition reduces circulating LBP levels in GF+WT mice during cholestatic disease

(A) ELISA analysis of serum samples obtained from vehicle/ GF+WT and ANIT/GF+WT mice Graph is representative of n=4-8 animals/treatment group (* P <0.05, ** P <0.01 [ANIT/GF+WT vs ANIT/clodronate/GF+WT and ANIT/MCC/GF+WT]).

SUPPLEMENTAL REFERENCES

1. Blokker BA, Maijo M, Echeandia M, Galduroz M, Patterson AM, Ten A, Philo M, et al. Fine-Tuning of Sirtuin 1 Expression Is Essential to Protect the Liver From Cholestatic Liver Disease. Hepatology 2019;69:699-716.
2. Hildebrand F, Tadeo R, Voigt AY, Bork P, Raes J. LotuS: an efficient and user-friendly OTU processing pipeline. Microbiome 2014;2:30.
3. Edgar RC. UPARSE: highly accurate OTU sequences from microbial amplicon reads. Nat Methods 2013;10:996-998.
4. Magoc T, Salzberg SL. FLASH: fast length adjustment of short reads to improve genome assemblies. Bioinformatics 2011;27:2957-2963.
5. Yilmaz P, Parfrey LW, Yarza P, Gerken J, Pruesse E, Quast C, Schweer T, et al. The SILVA and "All-species Living Tree Project (LTP)" taxonomic frameworks. Nucleic Acids Res 2014;42:D643-648.
6. Hildebrand F, Moitinho-Silva L, Blasche S, Jahn MT, Gossmann TI, Heuerta-Cepas J, Hercog R, et al. Antibiotics-induced monodominance of a novel gut bacterial order. Gut 2019.
7. Saary P, Forslund K, Bork P, Hildebrand F. RTK: efficient rarefaction analysis of large datasets. Bioinformatics 2017;33:2594-2595.
